# Endoscopic Transcanal Type 1 Tympanoplasty with Cartilage Perichondrium Graft; A Prospective Study 

**DOI:** 10.22038/ijorl.2025.83887.3826

**Published:** 2025

**Authors:** Parth Makwana, Viral Prajapati, Vivek Makadiya

**Affiliations:** 1 *Department of ENT, Nootan Medical College, Visnagar, Gujarat, India.*; 2 *Department of ENT, Dr. M. K.Shah Medical College, Ahmedabad, Gujarat, India.*

**Keywords:** Endoscopic tympanoplasty, Transcanal Tympanoplasty, Chronic Suppurative Otitis Media (CSOM), Tympanic Membrane Perforation

## Abstract

**Introduction:**

Recently use of endoscopes for ear surgeries is being popular among the surgeons as it gives a wide view of the middle ear. Also, tympanoplasty by transcanal approach can be done as a day care procedure and so it reduces the cost of hospitalization. The study was aimed to evaluate the results of type 1 tympanoplasty done by endoscopic transcanal approach with cartilage perichondrium graft, in relation to successful graft take-up rate and improvement in hearing.

**Materials and Methods:**

Total 50 patients were included in the study and all of them underwent type 1 tympanoplasty by endoscopic transcanal approach. Tragal cartilage with its attached perichondrium on one side facing laterally was placed as graft lateral to handle of malleus with v shaped notch at 12 o clock by underlay technique in all the cases. Patients were called for regular follow-up, and they were observed for successful graft take-up and improvement in hearing at 3 months after procedure.

**Results:**

Out of 50 cases complete closure was achieved in 46 cases which makes a success rate of 92%. The average improvement in hearing of 15.3 dB was noted.

**Conclusion:**

Endoscopic Transcanal approach with cartilage-perichondrium graft for tympanoplasty (type 1) is effective in terms of successful graft take-up rate and improvement in hearing, very cost effective, faster in terms of surgical time, and has less postoperative morbidity so gives better quality of life to the patient.

## Introduction

Chronic suppurative otitis media (CSOM) is defined as chronic inflammation of the mucosal lining of the middle ear cleft which may cause recurrent otorrhea, tympanic membrane perforation and decreased hearing. Tympanoplasty is defined as the surgical procedure for removing the disease from the middle ear, reconstructing the ossicular chain and repairing the tympanic membrane (TM) perforation by placing a graft. Mastoidectomy is needed along with tympanoplasty to remove the disease from the mastoid area. 

As the final step for the surgical correction of conductive hearing loss, tympanoplasty is done and it represents the summit of years of evolution for the surgical procedures done on the middle ear for hearing improvement (1). For tympanoplasty there are three surgical approaches: postaural, transcanal (transmeatal) and endaural. Each approach is having advantages and disadvantages, so no single approach is considered the best approach for all tympanic membrane perforations (2). The type of approach for tympanoplasty is decided on the factors like the site and size of the tympanic membrane perforation, size of the ear canal and experience and training of the surgeon. Recently the use of endoscopes for ear surgeries is being popular among the surgeons as it gives a wide view of the middle ear. Use of endoscopes for diagnosis and treatment of ear diseases started in 1960. In microscopic ear surgery for patients with cholesteatoma, endoscopes are used as support tools for disease clearance from hidden areas of middle ear and mastoid cavity. The first case of myringoplasty by endoscopic approach was reported in 1992 by El-Guindy (3). Subsequently, the reports for ear surgery by endoscopic approach were published by others like Raj and Meher in 2001, Yadav et al. in 2009, Ayache in 2013, and Dündar et al. in 2014 (4-7). Until 2009, successful graft uptake rate was reported between 80-90 percent; it has reached up to 96 percent recently. The reason for the same is that the resolution of the camera and monitor has increased. 

The use of endoscope for transcanal approach provides many advantages like: wide and clear view of the middle ear, attic and ossicles, minimal bleeding, Lesser or no complications; no post-operative bleeding or pain requiring treatment, cost effective, Faster recovery and faster healing, shorter hospital stay. Also, it can be done as day-care surgical procedure, Good Cosmetic results with approximately 1.5-2 cm healed scar on medial surface of tragus which is not visible and also disappears within few weeks, Possibility of performing surgery under local anesthesia, so can be performed in patients who are unfit for general anesthesia, significantly less surgical time. 

The disadvantages of endoscopic transcanal approach are: We have to work in a small external auditory canal, and the patients with hump in external auditory canal are difficult to perform the surgery, endoscope provides 2-D view, and the endoscope occupies one of the surgeon’s hands, there are chances of thermal injury and sensorineural hearing loss due to heat generation by light source, chances of trauma due to tip of endoscope and also by accidental movement of the patient during surgery and the instrumentation becomes overcrowded and also the instruments get tainted with blood sometimes and requires learning curve to overcome it. 

The study was aimed to evaluate the results of type1 tympanoplasty done by endoscopic transcanal approach with cartilage perichondrium graft, in relation to successful graft take-up rate and improvement in hearing. 

## Materials and Methods

This is an observational study, which was conducted at Dr. M. K. Shah Medical College & research Centre, Ahmedabad. 50 patients were included after obtaining the detailed informed consent, which outlined the procedure as well as its associated benefits and risks. 

Patients presenting at the Ear, Nose, and Throat (ENT) Outpatient Department (OPD) with symptoms of ear discharge and reduced hearing underwent thorough ENT examination. The criteria to include the patients in the study were as follows: Central perforation of the tympanic membrane - moderate to large in size, with mucosal type of chronic suppurative otitis media, having pure conductive type of hearing loss; dry ear status for more than 4 weeks; age over 18 years. Patients having ear discharge in the active stage were treated with appropriate local and systemic antibiotics to achieve a dry ear status for at least 4 weeks. The criteria to exclude the patients in the study were as follows: squamosal type of chronic suppurative otitis media, sensorineural type of hearing loss, intraoperative ossicular chain damage, Eustachian tube dysfunction, or nasal pathology identified during nasal endoscopy. 

Detailed history was taken from all the patients which was followed by thorough ENT clinical examination and all pertinent details were documented. Endoscopic evaluation of the ear was performed in each case using a zero-degree 4 mm high-definition endoscope and HD camera to assess size and location of tympanic membrane perforation and the condition of the middle ear mucosa. Central perforations affecting two or more quadrants of the pars tensa were considered for the study. 

Each eligible patient’s hearing was assessed preoperatively by pure tone audiometry (PTA) using a calibrated Audiometer. Air conduction (AC) threshold and bone conduction (BC) threshold were measured to assess the degree and type of hearing loss. Four frequencies (500 Hz, 1000 Hz, 2000 Hz, and 4000 Hz) were selected for the calculation of the average hearing loss (air-bone gap - ABG). Preoperative audiometry was conducted and then repeated three months after the procedure to assess changes in hearing loss. A comparison was made between the preoperative and postoperative audiograms to determine the extent of hearing improvement. 

The surgical procedure was performed under local anesthesia or general anesthesia as per patient’s comfort. The surgical procedure, guided by high-definition endoscopy, was conducted as follows: 

**Fig. 1A:**
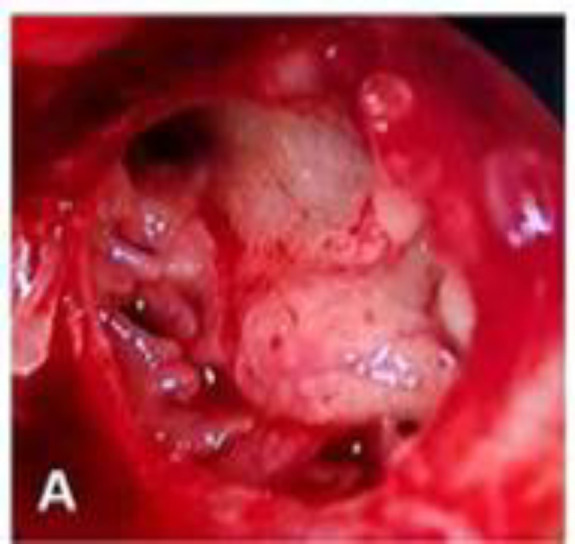
perforation magin freshening & TM undersurface debrided

The entire procedure was performed through a transcanal route with the use of a 4 mm sized zero-degree endoscope.

- The external auditory canal (EAC) was cleaned and 2% lignocaine with adrenaline was infiltrated in EAC.

- The tympanic membrane perforation margin was freshened and the medial surface of the TM was scraped with a micro-circular knife. (Fig. 1-A)

- A Rosen’s incision was kept approximately 5 mm lateral to annulus and with use of a circular knife the tympanomeatal flap was raised. (Fig. 1-B)

**Fig 1B:**
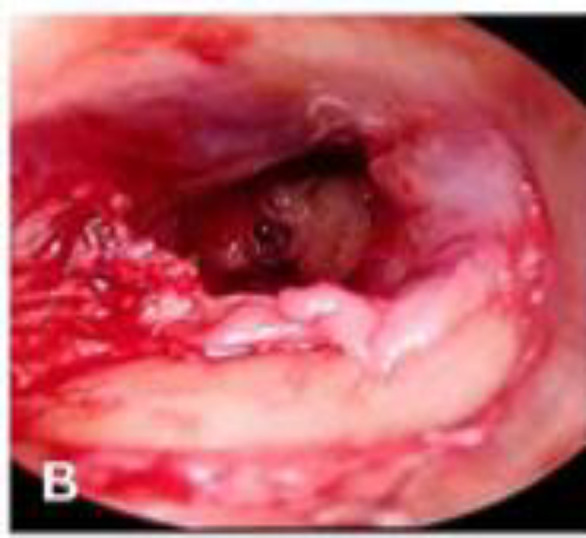
Rosen's incision kept & TM flap elevation

- The fibrous annulus was elevated from the tympanic sulcus and the ossicular chain continuity & mobility was checked.

- Incision was made on the skin over the medial aspect of tragus 2-3 mm medial to the lateral end of the tragus (Fig. 1-C).

**Fig 1C:**
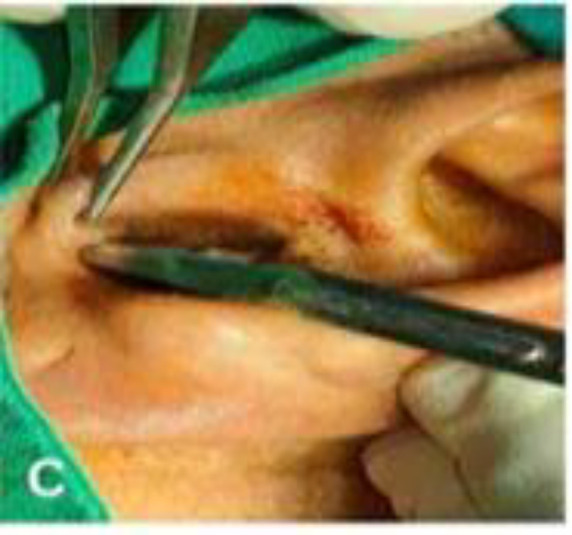
Incision being made on tragus

- The skin flap over tragus was then elevated and a tragal cartilage graft with perichondrium attached on one side was harvested. (Fig. 1-D)

**Fig 1D:**
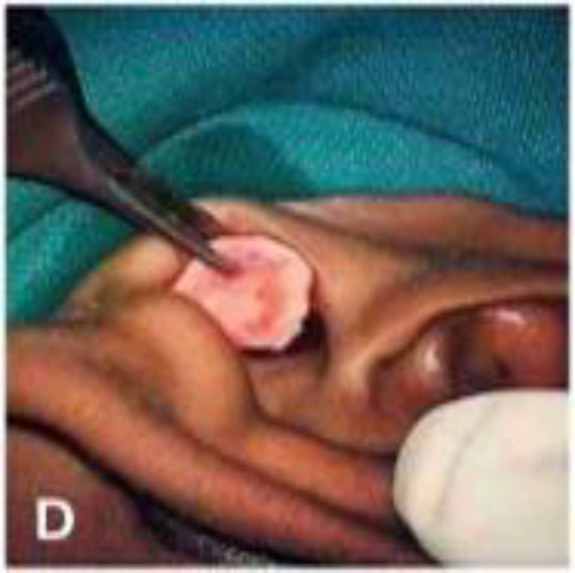
Harvesting Tragal cartilage with perichondrium

- The skin flap was reposited without using sutures.

- A small wedge-shaped notch was made at the 12 o'clock position and the graft was prepared according to the size of the perforation.

- The full thickness tragal cartilage graft with perichondrium attached on one side facing laterally was used for grafting.

- The graft which was prepared was placed lateral to the handle of malleus and medial to the fibrous annulus using underlay technique. (Fig. 1-E)

**Fig 1E:**
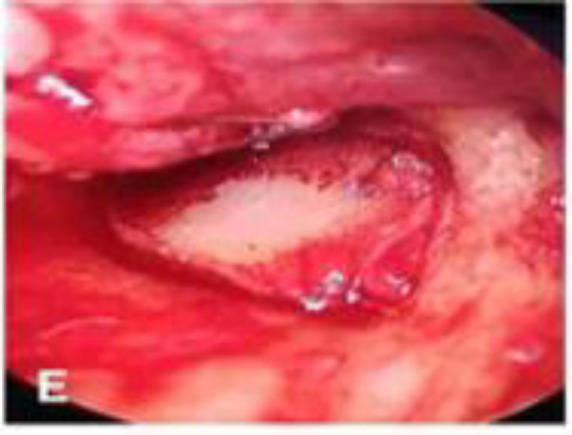
Graft placed lateral to Handle of Malleus

- The tympanomeatal flap was then repositioned (Fig. 1-F) and the external auditory canal was packed with antibiotic-soaked gel foam.

**Fig 1F:**
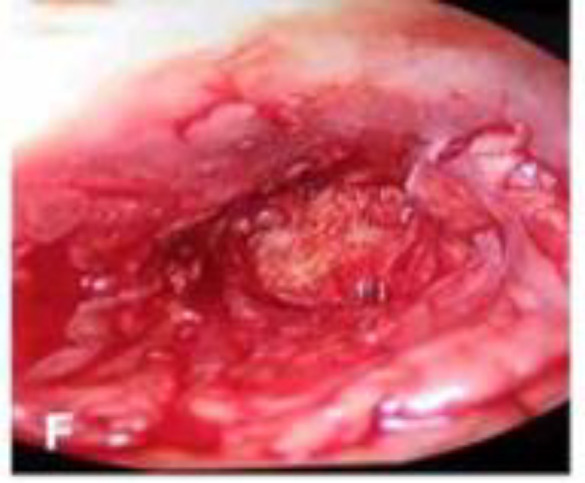
TM flap Reposited

- A small gauge wick was kept in the external auditory canal and ear bandage was given.

Patients were requested to come for follow-up examination at the end of 1st, 3rd, 6th and 12th week after the surgical procedure. Endoscopic examinations of ear and PTA examinations were done at 12 weeks after the surgical procedure and results were compared for graft take-up and improvement in hearing. On Endoscopic examinations of the ear at 12 weeks, cases with complete closure of perforation in tympanic membrane were labeled as successful graft uptake (Fig. 1-G). 

**Fig 1G:**
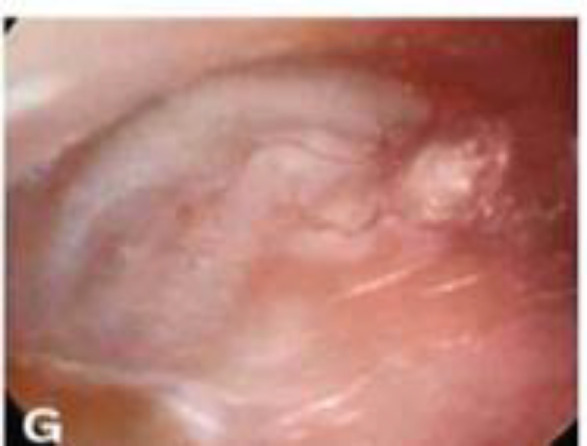
Successful graft uptake at 12 weeks

## Results

A total of 50 patients were included in the study, of which there were 28 male patients and 22 female patients. All the patients were between the ages of 18 to 60 years and the maximum of those (40 patients out of 50) were between 18 to 40 years. Out of these 50 patients, 39 patients were having unilateral disease and 11 were having bilateral disease. Out of the 50 patients, 46 patients acquired complete closure of the perforation with a success rate of 92%. 

In patients who achieved complete closure of perforation, the preoperative ABG was ranging from 20 – 45 dB and the postoperative ABG was ranging from 5 – 30 dB. Average ABG preoperatively was 30.65 dB and Average ABG postoperatively was 15.4 dB with average hearing gain was 15.3 dB. The 4 patients who did not have successful closure of perforation, one had fungal infection in the ear and the cartilage with perichondrium graft was completely dissolved and resulted in large perforation and the patient was subjected to Post aural tympanoplasty and cortical mastoidectomy with temporalis fascia graft. The rest 3 patients were having a minor space between the cartilage perichondrium graft and the margin of the perforation. They were subjected to conservative treatment as they did not have any complaints of ear discharge or hearing loss. 

## Discussion

Surgical repair of tympanic membrane perforation is routinely done by postaural approach and with temporalis fascia graft. With recent development of high-definition cameras and endoscopes the transcanal approach is becoming more and more popular. Also, the graft take-up rate and hearing improvement is also good with an endoscopic approach. An otologist can use the endoscope as a very versatile equipment. According to Fadl: “this approach merited the advantages of easier access to the middle ear, less bleeding, little scarring, timesaving and less subjected to infections” (8). The successful graft take-up rate in type I tympanoplasty by endoscopic approach was comparable to other studies that were previously reported which has increased because of increased resolution of camera and monitor, recently. 

Cartilage, perichondrium and fascia originate from mesenchymal tissue. Cartilage has long term viability, and it can be manipulated more easily than the fascia and it remains more stable during healing and prevents medialization of graft and prevents failure in early postoperative period. Cartilage accelerates healing of TM by way of epithelialization in the postoperative period (9, 10). The diffusion method gets tragal cartilage the required nourishment and eventually makes it incorporated into the tympanic membrane. Tragal cartilage is easily accessible, adequate in size and easy to manipulate intraoperatively and it can be harvested by a very small incision which is almost invisible postoperatively. Endoscopes being used more and more in ear surgery and tragal cartilage as graft material, the transcanal approach for ear surgeries is becoming very popular. 

There are various studies published related to endoscopic tympanoplasty and myringoplasty with different success rates. A comparison between such studies and present study is done and described in Table 1. In our study, we have used full thickness tragal cartilage as graft. As depicted in Table 1, our study also has achieved good graft take-up rate and good hearing improvement as compared to other studies. Type 1 tympanoplasty by endoscopic transcanal approach with cartilage graft is reliable and effective in patients with chronic suppurative otitis media. 

**Table 1:** comparison with other studies

**Sr. no.**	**Study by**	**Study Year**	**No. of Cases**	**Graft uptake Rate**	**Hearing Improvement in ABG (dB)**	**Follow-up Period**
1	Ayache (4)	2013	30	96%	-	1 Year
2	Huang et al (11)	2016	50	98%	8.9	6 months
3	Karhuketo et al (12)	2001	29	90%	<10 dB in 90% ears	1 Year
4	Mokbel et al (13)	2015	40	100%	8.50 +/- 1.25 dB	6 to 24 months
5	Singh et al (14)	2013	28	92.85%	15.65 dB	2 Years
6	Özgür et al (15)	2015	53	92.50%	10+/-7 dB	6 months
7	Vamanshankar (16)	2020	61	77%	10 dB	1 month
8	Shakya D (17)	2020	35	91.42%	9 dB	3 months
9	Parab S R. (18)	2020	69	97.1%	18.66 dB	1 Year
10	Parelkar K. (19)	2019	50	78%	14.08 dB	2 months
11	Present Study	2023	50	94%	15.3 dB	3 months

## Conclusion

Endoscopic Transcanal approach with cartilage-perichondrium graft for type 1 tympanoplasty provides good results in view of successful graft take-up rate and improvement in hearing. It is very cost effective, faster in terms of surgical time, and has less postoperative morbidity so gives better quality of life to the patient. 
